# Effects of a polyherbal formulation on the quality of life and survival of patients with common upper gastrointestinal cancers: A randomized placebo-controlled trial

**DOI:** 10.22038/AJP.2021.18132

**Published:** 2021

**Authors:** Azar Fani Pakdel, Ashkan Hatami, Roham Salek, Ali Taghizadeh-Kermani, Seyed Alireza Javadinia, Ahmad Ghorbani

**Affiliations:** 1 *Cancer Research Center, Mashhad University of Medical Sciences, Mashhad, Iran*; 2 *Surgical Oncology Research Center, Mashhad University of Medical Sciences, Mashhad, Iran*; 3 *Cellular and Molecular Research Center, Sabzevar University of Medical Sciences, Sabzevar, Iran *; 4 *Pharmacological Research Center of Medicinal Plants, Mashhad University of Medical Sciences, Mashhad, Iran*; 5 *Department of Pharmacology, Faculty of Medicine, Mashhad University of Medical Sciences, Mashhad, Iran*

**Keywords:** Camellia sinensis, Cancer, Curcuma longa, Allium sativum, Panax ginseng, Quality of life

## Abstract

**Objective:**

Previous clinical trials have suggested that herbal medicines can improve the quality of life (QOL) and survival of cancer patients. This study was aimed to evaluate the effects of a polyherbal compound (PHC, formulated as syrup) consisting of *Allium sativum*, *Curcuma longa*, *Panax ginseng*, and *Camellia sinensis* on the quality of life (QOL) and survival in patients with upper gastrointestinal cancers.

**Materials and Methods:**

A randomized placebo-controlled trial was carried out on patients with esophageal or gastric cancer who had finished their oncological treatments. The patients were randomly assigned to PHC (n=20) or placebo (n=20) group. The PHC group was treated with the PHC for 12 weeks, while the placebo group received 70% sucrose syrup. The QOL was assessed at baseline and after 12 weeks. The patients were followed for up to 24 months to determine overall survival.

**Results:**

PHC significantly improved cancer-related symptoms, physical performance, and psychological and social functions of the patients (p<0.05 for all cases). Death occurred in 33 and 22% of cases in the placebo and PHC group, respectively. The mean survival time was 16.8 months (95% CI: 12.8-20.9) in the placebo group and 21.4 months (95% CI: 19.1-23.6) in the PHC group but the difference was not statistically significant.

**Conclusion:**

The PHC improved cancer-related symptoms, physical performance, and psychological and social functions in patients with gastrointestinal cancers. It seems that this herbal compound has the potential to be used as a supplement in the management of cancer.

## Introduction

Cancers of the upper gastrointestinal tract, particularly gastric and esophageal cancers, are among the most common malignancies in the world. It is estimated that gastric cancer is the fourth leading cause of cancer death worldwide, accounting for 754000 deaths in 2015 (WHO, 2018 ▶). Similarly, esophageal cancer is one of the rapidly growing causes of cancer‐related deaths, as its incidence rate is expected to increase across high-income countries (Njei et al., 2016 ▶). Despite intensive chemotherapy, radiotherapy, and surgical interventions, a large number of patients with gastric and esophageal cancers have poor survival and quality of life (QOL) (Njei et al., 2016 ▶; Strong et al., 2015 ▶; Tantoy et al., 2018 ▶).

Herbal compounds have always been a good source for developing new drugs for different diseases. Beneficial effects of several medicinal plants have been shown in experimental and clinical studies for cancer chemoprevention/chemotherapy (Hosseini and Ghorbani, 2015 ▶; Jiao et al., 2018 ▶; Zaid et al., 2017 ▶). For many years, a number of herb-derived compounds such as vinca alkaloids, taxol analogs, and podophyllotoxin analogs have been used for chemotherapy of cancer patients (Saklani and Kutty, 2008 ▶). Based on a literature review (Hosseini and Ghorbani, 2015 ▶), four plants that their beneficial effects on cancer are supported by clinical trials, were chosen to prepare a polyherbal compound (PHC) for improving the QOL of cancer patients. These plants included *Allium sativum* (garlic), *Panax ginseng* (Asian ginseng), *Curcuma longa *(turmeric), and *Camellia sinensis* (green tea). It has been reported that *A. sativum* suppresses the size and number of adenomas in patients with colorectal adenomas (Tanaka et al., 2006 ▶). *P. ginseng* has inhibitory effects on cancer recurrence and improves postoperative survival in patients with gastric cancer (Suh et al., 2002 ▶). Also, in a cohort of breast cancer patients, a positive association has been found between ginseng use with survival and QOL (Cui et al., 2006 ▶). Similarly, it has been shown that green tea and curcuminoids (the active ingredients of turmeric) improve the QOL in patients with solid tumors (Bettuzzi et al., 2006 ▶; Panahi et al., 2014 ▶).

Multifactorial life-threatening diseases, for instance cancers, develop several complications and the treatment strategy usually consists of the use of different types of drugs. Hence, instead of mono-herb complementary therapy, the use of a combination of anti-cancer herbs may have a better effect on clinical outcomes. The present study was designed to evaluate the effects of a mixture of the above-mentioned herbs on the QOL and survival of patients with common upper gastrointestinal cancers.

## Materials and Methods

**Preparation of PHC**

The *A. sativum* L. clove (Alliaceae), *P. ginseng* Meyer root (Araliaceae), *C. longa *L. rhizome (Zingiberaceae), and *C. sinensis* L. leaf (Theaceae) were purchased from the division of Herbal Medicine of Imam-Reza Pharmacy (Mashhad, Iran). The hydroalcoholic extract of *C. longa* was prepared by macerating the plant powder (90 g) in 70% ethanol (900 ml) for 72 hr at 40ºC (Ghorbani et al., 2016 ▶). Then, the extract was ﬁltered through a 106 μm-mesh and concentrated to 100 ml (to remove ethanol) using a rotary vacuum evaporator (Stuart RE300, UK) at 70ºC. Hydroalcoholic extract of *C. sinensis* was prepared in a similar way to the *C. longa* extract. The juice of* A. sativum* cloves was obtained by homogenizing 45 g of the fresh cloves with 90 ml of distilled water in a juicer. To prepare a polyherbal syrup, 100 ml of *C. longa* extract, 100 ml of *C. sinensis* extract, 90 g of *P. ginseng* powder, and the juice of 45 g of* A. sativum* were mixed with 630 g honey and the volume was adjusted to 1800 ml with 70% sucrose solution. Therefore, each 20 ml of this PHC contains 35% (w/v) honey, the juice of 0.5 g of* A. sativum*, the extracts of 1 g of *C. longa* and 1 g of *C. sinensis*, and 1 g of *P. ginseng* powder. Bee honey was used in the formulation to reduce the unpleasant taste of* A. sativum* and *C. longa* and because it serves as an anticancer supplement that improves the QOL (Erejuwa et al., 2014 ▶; Singh, 2017 ▶).

**Standardizing PHC**

The PHC syrup was standardized based on its phenol content as determined by the Folin-Ciocalteu method (Hosseini et al., 2017 ▶). Briefly, a sample of 20 µl of the syrup was added to 100 µl of Folin-Ciocalteu reagent and 300 µl of sodium carbonate solution (1 mol/L). Then, the volume was brought to 2 ml with deionized water and the optical density was determined by a spectrometer at 765 nm. Gallic acid (0, 50, 100, 150, 250, and 500 mg/L) was used as the standard phenolic compound. The experiment was carried out in triplicate. The concentration of total phenols in the PHC syrup was 7.3±0.6 mg gallic acid equivalent per ml of the syrup. Since the patients were given the syrup at a volume of 20 ml three times daily, they received approximately 438 mg of the gallic acid equivalent of phenolic compounds each day.

**Ethics approval**

The study was conducted following the ethical standards of the institutional research committee and the 1964 Helsinki Declaration and its later amendments. The research protocol was approved by the ethics committee of Mashhad University of Medical Sciences, Iran (ethical code: IR.MUMS.REC.1394.598). The patients were informed about the aims of the study and informed written consent was obtained from all participants (Clinical trial registration: IRCT2017030416776N2).

**Inclusion/exclusion criteria**

Patients with gastric or esophageal cancers referred to the Oncology Clinic of Emam-Reza Hospital and the Oncology Clinic of Omid Hospital in Mashhad University of Medical Sciences were enrolled in this study. The inclusion criteria for enrollment were histologically proven gastric or esophageal cancer; completion of adjuvant therapy, and Eastern Cooperative Oncology Group performance status 0 or 1. The exclusion criteria were severe hepatic or renal damage, moderate-to-severe dysphagia, and distant metastasis. 

**Study design**

The study had a double-blind randomized placebo-controlled design. The patients were randomized by block randomization method with an allocation ratio of 1:1. For the primary outcome measure (global health status), the sample size was estimated based on data from a previous study (Kim et al., 2006 ▶), in which the mean of change in the total score of quality of life was 2.2±6.4 and 1.6±5.1 in the treatment (ginseng) and placebo group, respectively. Therefore, with a power of 80% and a significance level of 5%, it was calculated that a sample size of 36 per group would be needed (N= (1.96+0.84)^2^ (6.4^2^ + 5.1^2^) / [2.2 – (–1.6)]^2 ^=36). We estimated that about 9 months would be needed for collecting 72 patients but much fewer participants than expected met the inclusion criteria during this period. The registration period was extended for an additional 12 months. Despite this extension, we could not achieve the target sample size and decided to terminate the study with the 40 patients.

**Interventions**

The patients were randomly assigned to two groups: PHC (n=20) and placebo (n=20). The PHC group received the prepared syrup (20 ml, three times daily) for 12 weeks. Subjects in the placebo group received 70% sucrose syrup (containing edible red color) with the same schedule. The polyherbal and placebo syrups were packed in 250 ml bottles with the same shape and color and given hidden codes for the group assignment. The treating physician and the patients were blinded to the type of syrups.

**Assessments**

The health-related QOL, as the primary outcome, was evaluated using the Persian version of the European Organization for Research and Treatment of Cancer (EORTC) QOL Questionnaire (QLQ-C30 version 3.0), for which the reliability and validity were approved previously (Montazeri et al., 1999 ▶). The questionnaire consists of 30 items for assessing symptoms, functions, and global health status of cancer patients (Table 1). The items regarding symptoms and functions are 4-point scale (coded with responses “Not at all”, “A little”, “Quite a bit”, and “Very much”), while those related to global health status are 7-point scale items. Participants’ answers to all items were converted to a 0-100 scale using a standard scoring algorithm (Aaronson et al., 1993 ▶; Fayers et al., 2001 ▶). A low score for a functional scale or a high score for a symptom scale denote a high level of problem. Also, a low score for the global health status scale represents a low level of the QOL.

The secondary outcome was the overall survival of the patients, deﬁned as the time interval from the administration of PHC or placebo to death due to the disease.

**Statistical analysis**

The differences in the scores of the QOL between the placebo and treatment groups were statistically examined using an independent-sample *t*-test. Intragroup comparison of QOL scores between the baseline and after treatment was made by paired-sample *t*-test. Demographic data and categorized variables between the study groups were compared by the chi-square test. The Kaplan–Meier method was performed to compare overall survival, using the log-rank test to evaluate survival curves. Values are expressed as mean±standard deviation (SD) and a p-value of less than 0.05 was considered significant.

**Table 1:** The content of the European Organization for Research and Treatment of Cancer (EORTC) QOL Questionnaire (QLQ-C30 version 3)

Scope	Scale	Number of items	Item range
Functional scales	Physical functioning	5	3
	Emotional functioning	4	3
	Role functioning	2	3
	Social functioning	2	3
	Cognitive functioning	2	3
Symptom scales	Fatigue	3	3
	Pain	2	3
	Nausea and vomiting	2	3
	Appetite loss	1	3
	Constipation	1	3
	Diarrhea	1	3
	Sleep disturbance	1	3
	Dyspnea	1	3
	Financial difficulties	1	3
Global health status	Overall health and quality of life	2	6

Item range is the difference between the possible maximum and the minimum score to individual items (questions); most items take values from 1 to 4, giving a range of 3.

## Results

**Characteristics of the study groups**

**Figure 1:**
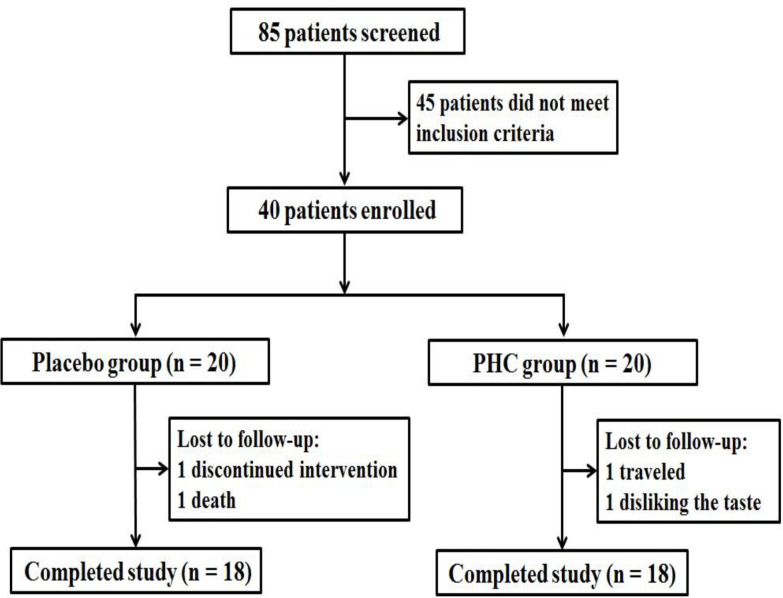
CONSORT diagram for patient recruitment and follow-up

Figure 1 shows the patient enrollment and randomization diagram. Of the 40 patients who were randomized in this trial, 36 completed the study: 18 in the placebo group and 18 in the PHC group. The demographic and cancer-related characteristics of the placebo and PHC groups are presented in Table 2. There were no significant statistical differences between the placebo and PHC groups in terms of age and cancer-related parameters (cancer location, histological diagnosis, and duration of cancer). Patients in the PHC group had a higher rate of female gender compared to the placebo group, but the difference was not statistically significant.

**Table 2:** The demographic and cancer-related characteristics of the patients who completed the study

Variables	Placebo group (n=18)	PHC group (n=18)	p-value
Age (years)	70±9	67±12	0.379
Gender Male (%) Female (%)	40 60	67 33	0.181
Cancer location Esophageal cancer (%) Gastric cancer (%)	60 40	72 28	0.506
Histological diagnosis Adenocarcinoma (%) Squamous-cell carcinoma (%)	43 57	31 69	0.707
Time since cancer diagnosis (months)	7±6	7±5	0.88

**Effect of PHC on QOL**

Mean scores for functional scales, symptom scales, and global health status scale at baseline and 12-week follow-up are presented in Table 3. At the baseline, there was no significant difference in any of the scales between the placebo and PHC patients. Among the patient symptoms, fatigue and appetite loss were the primary complaints. Also, they had relatively low physical and role functioning scores compared to other functions. Within-group analysis showed that treatment with PHC significantly improved fatigue (p<0.01), nausea and vomiting (p<0.01), appetite (p<0.05), constipation (p<0.05), diarrhea (p<0.05), global health status (p<0.01), and all functioning scales (p<0.05) except cognitive functioning. In the placebo group, only social functioning (p<0.05), appetite (p<0.01), and the global health status (p<0.01) were improved after 12 weeks. Between-group analysis showed greater improvement of role functioning, pain, and global health status in the PHC group compared to the placebo group (p<0.05).

**Survival analysis**

**Figure 2:**
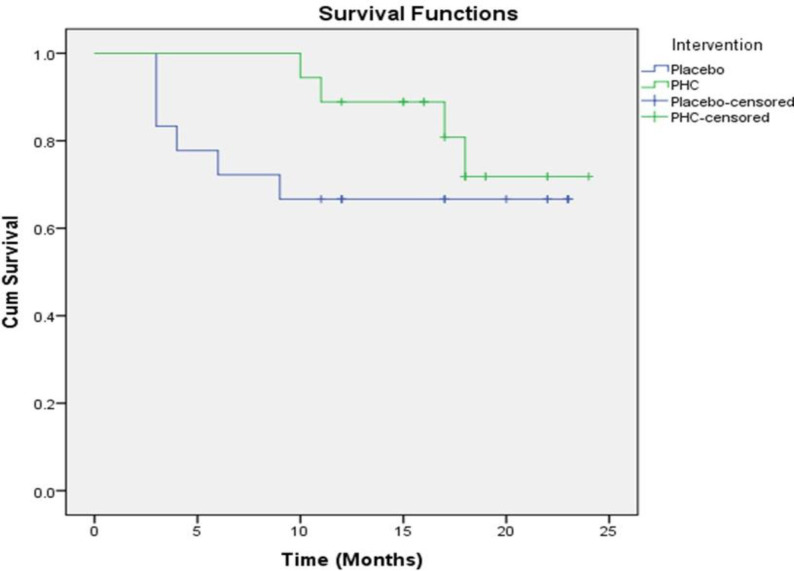
Kaplan-Meier curve of overall survival for patients treated for 12 weeks with the PHC or placebo

At the time of this writing, the median follow-up of patients was 17 months (range 3-24 months). During this follow‐up period, death had occurred in 6 of 18 (33.3 %) cases in the placebo group and 4 of 18 (22.2 %) cases in the PHC group. The mean survival time was 16.8 months (95% conﬁdence interval [CI] 12.8-20.9 months) in the placebo group and 21.4 months (95% CI 19.1-23.6 months) in the PHC group, but the difference was not statistically significant (p=0.317, log-rank test) (Figure 2).

**Table 3:** Effect of the formulated polyherbal compound (PHC) on the QOL-related outcomes in patients with common upper gastrointestinal cancers

QLQ-C30 scale	Placebo group		PHC group	
	Initial visit	12 week-visit	Initial visit	12 week-visit
Physical functioning	65.5±21.8	69.6±24	54.8±17.6	78.5±16.4***
Emotional functioning	72.2±19.3	77.7±24	66.6±30.5	81±18.4*
Role functioning	61.1±31.3	68.5±31.2	70.3±23.2	88.8±14**#
Social functioning	73.1±31.9	82.4±28.8*	71.2±29.5	90.7±18.2**
Cognitive functioning	82.4±19.3	80.6±18	80.5±17.3	87.9±14.9
Fatigue	42±26	32.7±16.8	43.8±21	21±20.8**
Pain	32.4±23.8	24±20.7	20.3±26.5	12±22#
Nausea and vomiting	22.2±26.8	5.5±16.2	22.2±21.3	1.8±5.3**
Appetite loss	46.3±34.5	17±26**	40.7±38.8	13±16.7*
Constipation	20.3±38	13±20.2	29.6±36	11.1±22.8*
Diarrhea	5.6±17	3.7±10.7	9.2±19	1.8±7.8*
Sleep disturbance	26±31.4	20.3±23.2	13±20.2	11.1±16.1
Dyspnea	20.3±28.3	13±28.3	16.6±28.5	5.5±12.7
Financial difficulties	24±27.5	16.6±28.5	29.6±32.1	18.5±28.5
Global health status	56.9±16.4	68±15.9**	64.8±20.3	80±13.1**#

Values are presented as mean score±SD. *p<0.05, **p<0.01, and ***p<0.001 compared to initial visit in the same group (Intragroup comparison); #p<0.05 compared to 12-week visit in the placebo group; QLQ-C30: European Organization for Research and Treatment of Cancer QOL Questionnaire.

## Discussion

The present study showed that PHC could significantly improve QOL and increase mean survival time from 16.8 to 21.4 months (though statistically non-significant). Although the survival of cancer patients has increased today (Jemal et al., 2017 ▶), a large number of the patients suffer from chemotherapy- and radiotherapy-related side effects that need to be managed to achieve a good QOL (Numico et al., 2015 ▶). In patients with gastrointestinal cancer, general symptoms affecting the QOL are fatigue, appetite loss, dysphagia, reflux, dyspnea, diarrhea, constipation, nausea, and vomiting (Derogar and Lagergren, 2012 ▶; Hwang et al., 2014 ▶; Karanicolas et al., 2013 ▶; Korenaga et al., 1992 ▶; Numico et al., 2015 ▶).

Previous clinical studies have suggested that complementary application of herbal compounds can reduce the side effects of standard chemo-/radiotherapies and may improve the QOL and survival of cancer patients (Bettuzzi et al., 2006 ▶; Cui et al., 2006 ▶; Hosseini and Ghorbani, 2015 ▶; Lenartz et al., 2000 ▶; Panahi et al., 2014 ▶; Piao et al., 2004 ▶; Suh et al., 2002 ▶). This study was designed to examine the effects of a herbal supplement consisting of four plants (*A. sativum*, *P. ginseng*, *C. longa*, and *C. sinensis*) on the QOL and survival in patients with common upper gastrointestinal cancers. The present data demonstrated that the formulated PHC was safe and effective for improving the functions, symptoms, and global health status of the patients. At the beginning of the study, patients in both groups had relatively low physical and role functioning scores and suffered from fatigue, pain, nausea, appetite loss, constipation, and sleep disturbance. Among these functions and symptoms, only appetite was improved in patients of the placebo group. A recent study evaluated changes in QOL scores of patients with gastrointestinal cancers over two cycles of chemotherapy and demonstrated that the scores of physical and mental domains of the QOL decreased in the first week following chemotherapy and then, increased in the following week (Tantoy et al., 2018 ▶). The present study was carried out on the patients who completed adjuvant therapy and the obtained results (placebo group) indicate that their global health status improved with time after adjuvant therapy. However, except for social functioning and appetite, other symptoms and functions did not change significantly over time in the placebo group. On the other hand, complementary treatment with PHC significantly improved fatigue, nausea, appetite loss, and constipation as well as the physical, emotional, social, and role functioning of the patients. Also, patients in the PHC group showed greater improvement in global health status compared to the placebo group. All components of the PHC might contribute to its beneficial effects on the QOL. It has been reported that green tea and curcuminoids of *C. longa* improve the QOL in patients with solid tumors (Bettuzzi et al., 2006 ▶; Panahi et al., 2014 ▶). In patients with breast cancer, administration of ginseng could improve the QOL (Cui et al., 2006 ▶). Also, it has been shown that bee honey can improve the QOL in patients with head and neck cancer (Singh, 2017 ▶). Apart from the QOL, the PHC may also induce an inhibitory effect on the tumor itself. This possibility should be tested in future works. However, previous studies have shown that the plants used in the PHC show anti-cancer effect (Tanaka et al., 2006 ▶). For example, *A. sativum* suppresses the size and number of adenomas in patients with colorectal cancer (Tanaka et al., 2006 ▶). Also, the plant extract exhibited anticancer stem cell activity against colon, prostate, breast, cervical, and hepatic cancer lines (Gore et al., 2021 ▶). Ginseng has inhibitory effects on cancer recurrence and improves postoperative survival in patients with gastric cancer (Suh et al., 2002 ▶). This plant was reported to facilitate the therapeutic effects of chemotherapy in patients with non-small cell lung cancer (Zhu et al., 2021 ▶). Green tea extract and some of its catechins suppress cell growth and induce apoptosis in prostate cancer (Chung et al., 2001 ▶). Furthermore, it has been shown that curcumin (the active ingredients of turmeric) analogues have anticancer effect on esophageal squamous cell carcinoma and gastric adenocarcinoma cells (Alibeiki et al., 2017 ▶).

Inhibition of cancer progression and increasing survival are the main goals of any anticancer therapy. In the present study, mortality in the PHC group was lower than that in the placebo group. Compared with the placebo, complementary treatment with the PHC could extend the overall survival by approximately 27%, although the difference was not statistically significant. A restriction of the present analysis is that the patients were followed up only up to 24 months and the number of events was relatively small. A further limitation is that our sample size was not large enough to permit a reliable subgroup analysis between patients with gastric cancer and those with esophageal cancer. A larger sample size could potentially bring about better results and therefore this study might be considered a pilot attempt in this field.

In conclusion, the results of this study indicate that complementary treatment with the PHC improves cancer-related symptoms, physical performance, and psychological and social functions in patients with common upper gastrointestinal cancers. Studies done during a longer period and on a larger number of patients are needed to confirm the beneficial effects of the formulated PHC.
